# Metagenomic Analysis of the Bacterial Community Associated with the Taproot of Sugar Beet

**DOI:** 10.1264/jsme2.ME14109

**Published:** 2015-02-14

**Authors:** Hirohito Tsurumaru, Takashi Okubo, Kazuyuki Okazaki, Megumi Hashimoto, Kaori Kakizaki, Eiko Hanzawa, Hiroyuki Takahashi, Noriyuki Asanome, Fukuyo Tanaka, Yasuyo Sekiyama, Seishi Ikeda, Kiwamu Minamisawa

**Affiliations:** 1 Graduate School of Life Science, Tohoku University2–1–1 Katahira, Aoba-ku, Sendai, Miyagi 980–8577Japan; 2 Hokkaido Agricultural Research Center, National Agriculture and Food Research Organization9–4 Shinsei-minami, Memuro-chou, Kasai-gun, Hokkaido 082–0081Japan; 3 Yamagata Integrated Agricultural Research Center, YamagataYamagata 990–2372Japan; 4 Agricultural Research Center, National Agriculture and Food Research Organization3–1–1 Kannondai, Tsukuba, Ibaraki 305–8666Japan; 5 National Food Research Institute, National Agriculture and Food Research Organization2–1–12 Kannondai, Tsukuba, Ibaraki 305–8642Japan

**Keywords:** *Bradyrhizobium*, metagenome, plant growth-promoting bacteria, quinoprotein glucose dehydrogenase, sugar beet

## Abstract

We analyzed a metagenome of the bacterial community associated with the taproot of sugar beet (*Beta vulgaris* L.) in order to investigate the genes involved in plant growth-promoting traits (PGPTs), namely 1-aminocyclopropane-1-carboxylic acid (ACC) deaminase, indole acetic acid (IAA), N_2_ fixation, phosphate solubilization, pyrroloquinoline quinone, siderophores, and plant disease suppression as well as methanol, sucrose, and betaine utilization. The most frequently detected gene among the PGPT categories encoded β-1,3-glucanase (18 per 10^5^ reads), which plays a role in the suppression of plant diseases. Genes involved in phosphate solubilization (*e.g.*, for quinoprotein glucose dehydrogenase), methanol utilization (*e.g.*, for methanol dehydrogenase), siderophore production (*e.g.* isochorismate pyruvate lyase), and ACC deaminase were also abundant. These results suggested that such PGPTs are crucially involved in supporting the growth of sugar beet. In contrast, genes for IAA production (*iaaM* and *ipdC*) were less abundant (~1 per 10^5^ reads). N_2_ fixation genes (*nifHDK*) were not detected; bacterial N_2_ -fixing activity was not observed in the ^15^N_2_ -feeding experiment. An analysis of nitrogen metabolism suggested that the sugar beet microbiome mainly utilized ammonium and nitroalkane as nitrogen sources. Thus, N_2_ fixation and IAA production did not appear to contribute to sugar beet growth. Taxonomic assignment of this metagenome revealed the high abundance of *Mesorhizobium*, *Bradyrhizobium*, and *Streptomyces*, suggesting that these genera have ecologically important roles in the taproot of sugar beet. *Bradyrhizobium*-assigned reads in particular were found in almost all categories of dominant PGPTs with high abundance. The present study revealed the characteristic functional genes in the taproot-associated microbiome of sugar beet, and suggest the opportunity to select sugar beet growth-promoting bacteria.

Microbial symbioses affect plant growth. For example, *Pantoea agglomerans* strain 33.1 was previously shown to promote the growth of sugarcane plants (37). Beneficial bacteria are termed “plant growth-promoting bacteria” (PGPB). To date, numerous studies have surveyed and characterized beneficial microbes among a wide variety of plant species (21, 27, 45, 52, 53). However, only limited success has been achieved in developing commercial microbial products for use as PGPB in agriculture. Most studies have screened PGPB based on the activities of plant growth-promoting traits (PGPTs), including 1-aminocyclopropane-1-carboxylic acid (ACC) deaminase, plant hormones such as indole acetic acid (IAA), N_2_ fixation, phosphate solubilization, pyrroloquinoline quinone (PQQ) production, siderophore production, and plant disease suppression (9, 34, 37, 51). However, since these traits have generally been investigated under laboratory conditions, it currently remains unclear whether the bacteria can function as PGPB under field conditions. Therefore, the isolation of PGPB for practical applications in agriculture requires time-consuming field trials, which impose a bottleneck in the practical application of PGPB to agriculture.

Sugar beet (*Beta vulgaris* L.) is the most important temperate crop in the commercial production of sucrose (13), and has drawn attention as a source of bioenergy (25). Although sugar beet produces more biomass than other temperate crops, the reason for this remains unclear. One possible explanation is its high affinity with beneficial microbes, as has also been reported in sweet potato (50). A recent bacterial community analysis by our group revealed that the taproots of sugar beet harbored a unique bacterial community dominated by the *Alphaproteobacteria*, including potentially beneficial groups such as *Bradyrhizobium* species (35).

Metagenomic analyses of plant-associated bacteria may not only help to explain the high productivity of sugar beet, but may also overcome the difficulties associated with surveying practical PGPB. For example, the frequent detection of phosphate solubilization-related genes suggests that the high productivity of sugar beet is supported by bacterial phosphate solubilization. In contrast, the infrequent detection of N_2_ fixation genes suggests that screening for N_2_ -fixing bacteria is not an efficient means for identifying promising PGPB. Thus, the characterization of genes underlying PGPTs in a metagenome of plant-associated bacteria may offer a realistic way to identify ideal beneficial bacteria in the screening.

As a first step toward elucidating the contribution of bacteria to the high productivity of sugar beet, we conducted a metagenomic analysis of the bacterial community associated with the taproot of sugar beet. We investigated not only typically known PGPTs, but also genes involved in the utilization of methanol, sucrose, and betaine; the application of methanol to plants can increase plant biomass, possibly due to the activity of methanol-utilizing bacteria (29), which has recently drawn attention (26). Sugar beet accumulates a large amount of betaine, as well as sucrose, in the taproot (28); therefore, the potential for utilizing these compounds may be a key function in the bacteria associated with the taproot of sugar beet. Only a few studies have conducted a metagenomic analysis of plant-associated bacteria (20, 40) due to the difficulty of avoiding contamination by host-plant genomic DNA (18). To the best of our knowledge, this is the first study to have performed a metagenomic analysis of the sugar beet-associated microbiome.

## Materials and Methods

### Plant materials and sampling

The seedlings of the sugar beet cultivar ‘Amahomare’ were grown in pots (paper pot no. 1; Nippon Beet Sugar Manufacturing, Tokyo, Japan) under greenhouse conditions between 16 March and 26 April 2010. They were then transplanted into an experimental field at the Hokkaido Agricultural Research Center (Memuro, Hokkaido, Japan, 42°53′32.5″N, 143°04′44.1″E, 94 m a.s.l.) on 26 April. Before transplanting, the field received 150 kg N as ammonium sulfate, 250 kg P_2_ O_5_ as calcium superphosphate, and 160 kg K_2_ O as potassium sulfate per hectare. On 12 July, visibly healthy sugar beets were randomly harvested. The taproots were carefully washed with tap water to remove adhering soil and organic debris and then rinsed with sterilized water. The lateral roots were manually removed with tweezers, and the taproots were stored at −30°C until use. The soil was also sampled at the time of sampling and its chemical characteristics were determined by the Tokachi Nokyoren Agricultural Research Institute (Obihiro, Hokkaido, Japan) (Table S1).

### Preparation of metagenomic DNA from taproot-associated bacteria on sugar beet

Each of the taproots was cut into several pieces. Bacterial cells, including both epiphytes and endophytes, were then directly extracted from ~200-g pieces without cultivation, as previously reported (18). This direct extraction of bacterial cells allowed for a large reduction in contamination by host-plant genomic DNA. Cells were extracted from nine taproot samples. To obtain a sufficient amount of metagenomic DNA, a composite sample was made by combining extracts derived from three samples of taproots, giving three composite samples for subsequent experiments. Metagenomic DNA was prepared from each of these three composite samples according to a previously reported method (18).

### Metagenomic analysis of the bacterial community associated with the sugar beet taproot

The metagenomic DNAs (*n*=3) of the bacterial community associated with the taproots of sugar beet were sequenced by a next-generation sequencer (454 GS FLX+; Roche Diagnostics, Tokyo, Japan). The raw sequences were not assembled. Potentially contaminated sequences derived from the host plant genome (approximately 2% of the raw sequence reads), which were assigned as *Streptophyta* by the best BLASTN (2) hit against the NCBI non-redundant nucleotide database, were removed. These sequence reads were analyzed by a BLASTX search (2) against the NCBI non-redundant protein database (which was downloaded in December 2012 and contains 21,985,448 sequences) with an E-value cut-off of 10^−10^. The BLASTX results were imported into MEGAN software (version 4.70.4) (17), which then analyzed potential functional genes by both the KEGG (Kyoto Encyclopedia of Genes and Genomes; http://www.genome.jp/kegg/) and SEED classification systems (http://www.theseed.org/wiki/Home_of_the_SEED) (36). The genes were taxonomically assigned on the basis of the best BLAST hit as provided by the inspector tool in the MEGAN software. A rarefaction curve of the metagenomic DNA samples was described using the Metagenomics RAST server (MG-RAST; http://metagenomics.anl.gov) (31).

We investigated the genes underlying PGPTs, N metabolism, and the utilization of methanol, sucrose, and betaine. The PGPTs consisted of ACC deaminase, IAA, N_2_ fixation, phosphate solubilization, PQQ biosynthesis (*pqqC*), siderophores, and plant disease suppression. Regarding methanol utilization, we examined methanol dehydrogenase (MDH), which mediates the first step in C1 metabolism. The two known types of MDH genes (*xoxF* and *mxaF*) could not be distinguished by a simple BLAST or MEGAN analysis owing to high sequence similarities. As an example of high similarity, the amino acid sequence similarities between *xoxF* and *mxaF* genes in *Methylobacterium extorquens* AM1 are shown in Fig. S1. Therefore, we conducted a phylogenetic tree analysis to discriminate them by phylogenetic distance (Fig. S2), as shown by Jewell *et al.* (22) and Kalyuzhnaya *et al.* (23). The phylogenetic tree was created by the neighbor-joining method in MEGA6 software (http://www.megasoftware.net) (49). Since the genes for N_2_ fixation, methanol utilization, and the PQQ biosynthesis protein were not detected by the KEGG and SEED databases in MEGAN, we reanalyzed the metagenome by TBLASTN (E-value <10^−10^, hit length >250 bp, identity >40%) using the following amino acid sequences as queries: *nifHDK* in *Bradyrhizobium japonicum* USDA110; *pqqC* in *Gluconacetobacter diazotrophicus* PAl5 and *Pseudomonas fluorescens* SBW25; and *xoxF* and *mxaF* in *Methylobacterium extorquens* AM1. The RefSeq numbers for *nifHDK* in *B. japonicum* USDA110 were NP_768409, NP_768383 and NP_768384, respectively, those for *pqqC* in *G. diazotrophicus* PAl5 and *P. fluorescens* SBW25 were YP_001601525 and YP_002875095, respectively, and those for *xoxF* and *mxaF* in *M. extorquens* AM1 were YP_002962861 and YP_002965446, respectively.

The frequency of detection of potential functional genes was calculated as follows: Frequency = (Number of sequence reads for a target gene/Gene length) × (100,000/Total number of sequence reads). The detected number of target genes was normalized by the gene length (kbp). An approximate gene length (kbp) was deduced by using the information of the UniProt Reference Clusters (UniRef) database (http://www.uniprot.org/uniref/). The detection frequency, normalized by gene length (kbp), was expressed per 10^5^ reads by multiplying (100,000/Total number of sequence reads). The “Total number of sequence reads” of the metagenomic DNA samples (samples NPK9, NPK10, and NPK12) is shown in Table 1. A taxonomic assignment analysis of all sequences was performed by MEGAN, and relative abundances were also calculated based on the average of three metagenome samples.

### ^15^N_2_ exposure experiment with sugar beet root

The seedlings of the sugar beet cultivar ‘Rycka’ were grown in pots as described above under greenhouse conditions between 14 March and 2 May 2012. They were then transplanted into an experimental field at the Yamagata Integrated Agricultural Research Center (Yamagata, Japan, 38°14′56.8″N 140°14′39.6″E, 225 m a.s.l.) on 2 May. Before fertilizer was applied, a soil sample was taken and chemically characterized (Table S1). Before transplanting, the field received 150 kg N as ammonium sulfate, 210 kg P_2_ O_5_ as calcium superphosphate, and 140 kg K_2_ O as potassium chloride per hectare. Since the values of δ^15^N-air (‰) may be affected by the presence of different nitrogen sources (ammonium sulfate and soil nitrogen), we also included a field on which ammonium sulfate was not applied. On 12 July, healthy sugar beet plants were harvested. After washing in tap water, the aerial part was cut off. The taproots were then transferred into a plastic box (SB-II; Sanplatec, Osaka, Japan). The air in the box was replaced with ^15^N_2_ /O_2_ /Ar gas (35:3:62 [v/v/v]; 99.4 atom % ^15^N; Shoko, Tokyo, Japan), and it was incubated for 24 h at 25°C. Unexposed taproots were used as a negative control. The δ^15^N-air content of the roots was analyzed by SI Science (Saitama, Japan) on an elemental analyzer/isotope ratio mass spectrometer (EA/IRMS) (Flash EA1112-DELTA V ADVANTAGE ConFlo IV System; Thermo Scientific, Tokyo, Japan). The δ^15^N-air values of the N sources (ammonium sulfate and soil before cultivation) were also analyzed. The significance of the difference in δ^15^N-air between ^15^N_2_ -exposed and unexposed taproots was determined by the *t*-test (*n*=3, *P*<0.05).

### Accession number of metagenomic DNA sequences

The metagenomic DNA sequences analyzed in the present study were deposited in the DDBJ Sequence Read Archive (accession no. DRA000977).

## Results

### Statistical summary of metagenomic data

The metagenomic DNA samples (*n*=3; samples NPK9, NPK10, and NPK12 in Table 1) were sequenced, resulting in a total of 183,754 sequence reads. The average read length of the three samples was 645 bp (Table 1). A BLASTX search showed that 54% of the total sequence reads (100,053 reads) had similarities (E-value <10^−10^) to sequences in the NCBI non-redundant protein database. A rarefaction curve of the metagenomic DNA samples is shown in Fig. S3.

### Detection frequency of potential functional genes in the metagenome

A MEGAN survey found most of our target functional genes (Table 2), except for those related to N_2_ fixation (*nifHDK*), PQQ biosynthesis (*pqqC*), and methanol utilization (MDH). A reanalysis by TBLASTN search also did not detect *nifHDK*, but did detect *pqqC*- and MDH-related sequences. To determine the type of MDH (*xoxF* or *mxaF*), we measured the phylogenetic distance; however, the MDH type of most sequence reads (22/34 reads) could not be allocated (Table S2) because these reads were clustered with the out-group. Furthermore, a phylogenetic analysis with multiple sequence reads was not successful due to the high sequence dissimilarity. Therefore, we analyzed the MDH-related sequences one by one. An example result with one sequence read (HSSJVCC01DDEZ5 in sample NPK9) is shown in Fig. S2. This read was located in a cluster of *xoxF*-type genes. Interestingly, only *xoxF*-type genes were present in the microbiome (Table S2).

Because the KEGG classification system included multiple types of genes for siderophores, only the three most abundant genes are shown (Table 2) (The full set is shown in Table S3). Isochorismate pyruvate lyase (*pchB*) was the most abundant (10 per 10^5^ reads) among the genes for siderophores.

Among the categories of PGPTs and methanol utilization, β-1,3-glucanase was the most frequently detected (18 per 10^5^ reads), followed by quinoprotein glucose dehydrogenase (GDH), MDH, isochorismate pyruvate lyase (*pchB*), ACC deaminase, and chitinase (15, 10, 10, 9, and 6 per 10^5^ reads, respectively) (Table 2). A taxonomic assignment analysis revealed that some of the β-1,3-glucanase genes were assigned to the genus *Bradyrhizobium* with high abundance (Table S4). The high proportion of *Bradyrhizobium*-assigned sequences was also observed in other abundant PGPT genes, except for the *pchB* gene (Tables S5–S9). Meanwhile, IAA-related sequences (*iaaM* and *ipdC*) were detected at a low frequency (0.7 and 0.8, respectively, per 10^5^ reads) while genes for N_2_ fixation (*nifHDK*) were not detected as described above.

In the categories of sucrose and betaine utilization, genes related to glycine betaine/proline transport systems were relatively abundant (13–25 per 10^5^ reads; Table 2). However, the gene related to betaine-homocysteine S-methyltransferase, which is involved in the degradation of betaine, was uncommon (0.8 per 10^5^ reads).

Since we failed to detect any N_2_ fixation genes, we surveyed genes for N metabolism. The KEGG classification system includes multiple types of N-metabolism-related genes; therefore, only the eight most abundant genes are shown in Table 2 (results with the full set of genes are shown in Table S10). In this category, the most frequently detected genes were involved in the glutamine synthetase-glutamate synthase (GS-GOGAT) pathway: *glnA*, *gudB*, *gltD*, and *gltB* (49, 26, 20, and 16 per 10^5^ reads, respectively). These genes catalyze a series of enzymatic reaction steps that convert ammonium to glutamate. We also frequently detected genes for nitronate monooxygenase (*ncd2*; 31 per 10^5^ reads) and nitrite reductase (*nirB*; 11 per 10^5^ reads), which catalyze the conversion of nitroalkane to ammonium via nitrite.

### ^15^N abundance in sugar beet taproots

The values (means ± standard deviations) of δ^15^N-air in the taproots with and without ammonium sulfate fertilizations were −6.083±0.336‰ and 2.169±0.261‰, respectively (Fig. S4). The reduction of δ^15^N by the application of the fertilizer was due to the lower value of δ^15^N for the fertilizer (−9.863‰) than for the original soil (5.477‰) (Fig. S4). The values of δ^15^N-air in the taproots exposed to ^15^N_2_ gas with and without ammonium sulfate fertilizations were −5.501±0.717‰ and 2.198±0.213‰, respectively. No significant difference was observed in δ^15^N-air values between ^15^N_2_ -exposed and unexposed taproots with and without ammonium sulfate fertilization (Fig. S4). Thus, the bacterial fixation of atmospheric N_2_ was not detected.

### Relative abundance of major taxonomic groups

At the class level, the *Alphaproteobacteria* were dominant (72%), followed by the *Actinobacteria* (10%) and the *Betaproteobacteria* (8%) (Table 3). Within the *Alphaproteobacteria*, the orders *Rhizobiales* (55%) and *Sphingomonadales* (14%) were dominant. At the family level, the *Bradyrhizobiaceae* (26%), the *Rhizobiaceae* (17%) and the *Sphingomonadaceae* (11%) were highly abundant. At the genus level, *Mesorhizobium* (14%), *Bradyrhizobium* (11%), and *Streptomyces* (9%) were the most predominant.

## Discussion

We analyzed potential functional genes for PGPTs and the utilization of methanol, sucrose, and betaine in the bacterial community of the taproot of field-grown sugar beet (Table 2). Among the categories for PGPTs and methanol utilization, the gene for β-1,3-glucanase was the most frequently detected (18 per 10^5^ reads; Table 2). Genes for chitinase, isochorismate pyruvate lyase (*pchB*), and quinoprotein glucose dehydrogenase (GDH) were also detected (6, 10, and 15 per 10^5^ reads; Table 2). These genes are involved in the suppression of plant diseases. Genes for β-1,3-glucanase and chitinase are well known for their antibiotic activities against certain microbial groups (10, 32), and the combination of β-1,3-glucanase and chitinase has been shown to very effectively control fungal diseases (30). The siderophore produced by the *pchB* gene is also known to be involved in disease suppression because plant pathogens can be suppressed through an iron deficiency by chelating iron with siderophores (1, 4). Gluconic acid produced by GDH, as described below, also has antifungal activity (24).

The gene for GDH catalyzes the production of gluconic acid from glucose, and aids phosphate solubilization (3). This reaction has been referred to “direct glucose oxidation” in order to distinguish it from oxidation via the pentose phosphate pathway (12). The abundance of GDH genes (15 per 10^5^ reads) is consistent with the high ability of sugar beet to adapt to phosphate deficiency (46, 47).

Genes encoding methanol dehydrogenase (MDH) were detected at 10 per 10^5^ reads (Table 2). A phylogenetic analysis revealed that they were exclusively affiliated with *xoxF* (Table S2), which suggests the ecological importance of *xoxF* for plant-associated bacteria in the taproot of sugar beet, as previously indicated in the aerial parts of soybean, clover, and *Arabidopsis* (11). The potential ecological advantage for methylotrophic bacteria in the rhizosphere has been reported (48). Thus, C1 metabolism, notably methanol utilization, appears to be an important function for microbial communities in the rhizosphere, as in the phyllosphere (11). The activities of XoxF and MxaF require La^3+^ and Ca^2+^ as co-factors, respectively (33). Some strains of *Bradyrhizobium* sp., isolated from the sugar beet taproot in a previous study (35), grew with methanol as a carbon source in the presence of La^3+^ (data not shown). We detected a trace amount of methanol in the taproots of sugar beet (data not shown). Since methanol is released during the microbial degradation of pectin (the backbone of plant cells) (39), C1 metabolism may be important for the environmental fitness of plant-associated microbes. Further experiments are needed in order to elucidate the ecological role of *xoxF* in plant root-associated bacteria.

The detection frequency of the genes for ACC deaminase and PQQ biosynthesis (*pqqC*) were 9 and 3 per 10^5^ reads. ACC deaminase promotes plant growth by decreasing ethylene in plants, which has been shown to inhibit plant growth (51). PQQ is required for the activities of GDH and MDH, both of which were more abundant (15 and 10 per 10^5^ reads) than PQQ (3 per 10^5^ reads) in the metagenome of taproot-associated bacteria on sugar beet (Table 2). The imbalance observed in gene detection frequency may have been due to sugar beet-associated bacteria absorbing PQQ from the surrounding environment as a vitamin, as suggested by Goosen *et al.* (14) and Babukhan *et al.* (3).

The abundance of genes for IAA production (*iaaM* and *ipdC*) was lower (~1 per 10^5^ reads) than those for disease suppression, phosphate solubilization, methanol utilization, and ACC deaminase. Genes for bacterial N_2_ fixation were not detected by the metagenomic analysis (Table 2). Sugar beet requires larger amounts of N fertilizer (8, 35) than other major crops (barley, soybean, or rice plants) (8, 18, 19); therefore, the larger amount of N applied (150 kg N ha^−1^) may have affected the diversity and functionality of the sugar beet microbiome. However, N_2_ fixation activity was not detected in the ^15^N_2_ -feeding experiment (Fig. S4) even when sugar beet was cultivated in a field on which N fertilizer had not been applied (the contents of NH_4_ -N and NO_3_ -N in this soil were 272 and 149 mg kg^−1^, respectively; Table S1). The lack of N_2_ fixation ability was unexpected because diazotrophic bacteria have frequently been found in sugar-accumulating plants and are some of the most common functional members of the bacterial community in diverse environments (50, 52). An analysis of N metabolism suggested that the bacteria associated with the taproot of sugar beet were able to utilize ammonium or nitroalkane as a nitrogen source (Table 2). In N metabolism, the *glnA* gene for glutamine synthetase was the most abundant. This gene catalyzes an enzymatic reaction that converts ammonium to glutamine. Glutamine has been identified as the major amino acid (approximately 40% in amino N) in sugar beet (16).

The results shown in Table 2 suggest that a significant portion of the high biomass productivity of sugar beet is attributable to the high abundance of genes for plant disease suppression, phosphate solubilization, methanol utilization, and ACC deaminase in the microbiome. These results also showed that the screening of bacterial traits for N_2_ fixation and IAA production may not be an efficient means for surveying growth-promoting bacteria. In previous studies, growth-promoting bacteria for sugar beet were isolated on the basis of N_2_ fixation and IAA production (7, 8, 38, 42, 43). These differences between the present and previous studies may be explained by the different target tissues analyzed; we examined the taproots and excluded the lateral roots, which could gain considerable benefit for its extensive growth from microbial IAA production in a rhizosphere (15). Meanwhile, the direct contribution of bacterial N_2_ fixation to plant growth promotion currently remains unclear. Other PGPTs besides N_2_ fixation may be more important in plant growth promotion than previously thought, as demonstrated by the *nifH* mutant of a diazotrophic endophyte promoting sugarcane growth to the same extent as the wild type (6, 41).

In the categories for sucrose and betaine utilization, genes related to glycine betaine/proline transport systems were relatively abundant (13–25 per 10^5^ reads; Table 2). However, genes for betaine-homocysteine S-methyltransferase, which degrades betaine, had low abundance (0.8 per 10^5^ reads). These results suggest that bacteria associated with the taproot of sugar beet utilize betaine as an osmotic pressure regulator, but not as a nitrogen or carbon source. Some strains of *Bradyrhizobium* species, isolated from the sugar beet taproot (35), were not able to utilize betaine as a carbon source (data not shown).

The ability to colonize plants is an important trait for PGPB, as shown in studies characterizing the tissue localization of PGPB (37). Therefore, phylogenetic abundance in a metagenome of specific plant tissues may be a reasonable indicator for surveying potential PGPB. *Alphaproteobacteria* were the dominant taxonomic group in the microbiome of the taproot of sugar beet (Table 3). This result is consistent with previous findings reported by our group (35) and Shi *et al.* (44), who evaluated bacterial diversity based on 16S rRNA gene sequence analyses. In our previous study, we isolated 531 strains in 155 operational taxonomic units at the species level from the taproots of sugar beet to construct a bacterial collection as a resource for PGPB screening (35). Three genera (*Mesorhizobium*, *Bradyrhizobium*, and *Streptomyces*) were identified as an abundant taxonomic group in both the previous and present studies (Table 3). In addition, the dominant genes examined in the present study (underlying plant disease suppression, phosphate solubilization, methanol utilization, and ACC deaminase) were frequently assigned to these genera (Tables S4–S6 and S8–9). Therefore, these abundant bacterial groups may be potentially important candidates as PGPB with their high affinity for the taproot of sugar beet and have beneficial functions for plant growth promotion. Since *Bradyrhizobium*-assigned reads in particular were abundantly found in all categories (except for the *pchB* gene) of dominant PGPTs (Tables S4–S9), *Bradyrhizobium* species isolated from the taproot of sugar beet may be promising candidates for PGPB.

In the present study, we analyzed functional genes, potentially involved in PGPTs (Table 2), in the metagenome of taproot-associated bacteria on sugar beet. Further studies need to be conducted using gene expression and/or proteome analyses under field conditions, as described previously for the rice plant (5), in order to clarify the ecological roles of the abundant genes for PGPTs.

Comprehensive screening and genome analyses of PGPB for sugar beet are now ongoing based on the results of the present and previous studies conducted by our group (35). These efforts may lead to the identification of a novel mechanism that supports the high productivity of sugar beet and provide an efficient means to survey practical PGPB for agricultural practices.

## Supplementary Information



## Figures and Tables

**Table 1:** Statistical summary of metagenomic data obtained from the bacterial community of sugar beet taproot

	Sample name			Sum

	NPK9	NPK10	NPK12	
Total number of sequence reads	46,605	57,341	79,808	183,754
Sequence length (bp)	29,467,018	37,031,295	52,179,838	118,678,151
Average sequence length (bp/read)	632	645	653	645
Number of reads showing a similarity to sequences in the NCBI nr protein database^a^	25,914	31,528	42,611	100,053

a The BLASTX program (e-value cut-off <10^−10^) was used in the similarity search.

**Table 2:** Detection frequency of potential functional genes in the bacterial community of sugar beet taproot

	ID of KEGG	Gene length^a^		Frequency per 10^5^ reads^b^

		ID of UniRef	length (kbp)	
**ACC deaminase**	K01505	UniRef50_A5EJ46	1.020	**9±6**
**IAA**				**1±0.5**
tryptophan 2-monooxygenase (*iaaM*)	K00466	UniRef50_A6W7Y1	1.707	0.7±0.5
indolepyruvate decarboxylase (*ipdC*)	K04103	UniRef50_I0BL41	1.755	0.8±0.6
**N_2_** **fixation**^c^				**ND**
nitrogenase iron protein (*nifH*)	K02588	UniRef50_O07641	0.984	ND
nitrogenase molybdenum-iron protein alpha chain (*nifD*)	K02586	UniRef50_P19066	1.515	ND
nitrogenase molybdenum-iron protein beta chain (*nifK*)	K02591	UniRef50_P25314	1.566	ND
nitrogenase delta subunit (*anfG*)	K00531	UniRef50_O68940	0.351	ND
**PQQ biosynthesis protein (*pqqC*)**^d^		UniRef50_Q6F9J1	0.768	**3±0.4**
**Phosphate solubilization**				**19±6**
quinoprotein glucose dehydrogenase (GDH)	K00117	UniRef50_P27175	2.427	15±5
3-phytase	K01083	UniRef50_G2IPZ8	1.074	3±2
4-phytase	K01093	UniRef50_B0UQX3	1.635	0.3±0.4
**Siderophore**^e^				**18±9**
isochorismate pyruvate lyase (*pchB*)	K04782	UniRef50_Q51507	0.306	10±7
nonribosomal peptide synthetase (*dhbF*)	K04780	UniRef50_C6U462	3.270	3±0.6
enterobactin synthetase component F (*entF*)	K02364	UniRef50_P11454	3.882	1±0.6
other genes				4±3
**Plant disease suppression**				**24±8**
β-1,3-glucanase	K01210	UniRef50_R4MQJ4	0.885	18±6
chitinase	K01183	UniRef50_I4XS16	1.464	6±4
**Methanol utilization (Methanol dehydrogenase (MDH))**^f^				**10±3**
undetermined MDH		UniRef90_C5ATJ3^g^	1.800	6±0.8
*xoxF* gene type		UniRef90_C5ATJ3	1.800	3±2
**Sucrose utilization**				**7±1**
*sacA*	K01193	UniRef50_P07819	1.440	6±2
PTS system, sucrose-specific IIA component	M00269	UniRef50_S6C6M4	1.968	0.6±0.9
**Betaine utilization**				**54±8**
glycine betaine/proline transport system (*proX*)	K02002	UniRef50_P0AFM3	0.993	25±3
glycine betaine/proline transport system (*proW*)	K02001	UniRef50_P17327	1.065	13±4
glycine betaine/proline transport system (*proV*)	K02000	UniRef50_P14175	1.203	16±3
betaine-homocysteine S-methyltransferase	K00544	UniRef50_A4WQF1	1.074	0.8±1
**N metabolism-related genes**^g^				**296±22**
glutamine synthetase (*glnA*)	K01915	UniRef50_A0R079	1.437	49±6
carbonic anhydrase (*cynT, can*)	K01673	UniRef50_Q9I262	0.663	40±6
nitronate monooxygenase (*ncd2, npd*)	K00459	UniRef50_F8GQA6	1.254	31±4
glutamate dehydrogenase (*gudB, rocG*)	K00260	UniRef50_B2RKJ1	1.338	26±2
glutamate synthase (NADPH/NADH) small chain (*gltD*)	K00266	UniRef50_P9WN18	1.467	20±3
glutamate synthase (NADPH/NADH) large chain (*gltB*)	K00265	UniRef50_Q05755	4.548	16±3
nirnitrite reductase (NO-forming) (*nirK*)	K00368	UniRef50_P81445	0.993	13±8
nitrite reductase (NADH) large subunit (*nirB*)	K00362	UniRef90_A6UI45	2.463	11±8
other genes				91±17

a Approximately gene length (kbp) was deduced by using the information of the UniProt Reference Clusters (UniRef) database.

b ND = not detected. The values represent means ± standard deviation (*n*=3).

c *nifHDK* genes were not found by both MEGAN and TBLASTN search.

d *pqqC* gene was not found by MEGAN, but found by TBLASTN search.

e Top three abundant genes were shown. Other genes were grouped in the ‘other genes’ category. Full list of the genes for siderophore were shown in Table S3.

f MDH gene was not found by MEGAN, but found by TBLASTN search. Type of MDH (*xoxF* or *mxaF*) was determined by the phylogenetic tree analysis (Table S2). In almost sequence reads, the MDH type could not be determined because these sequence reads located in the out group of *xoxF* and *mxaF* phylogenetic tree. Such sequence reads were shown as “undetermined MDH”.

g Top eight abundant genes were shown. Other genes were grouped in the ‘other genes’ category. Full list of nitrogen metabolism related genes were shown in Table S10.

h The same ID No. of UniRef as *xoxF* gene was used.

**Table 3:** Relative abundance of major taxonomic groups in the bacterial community of sugar beet taproot

Major taxonomic groups^a^	Relative abundance (%)^b^
**Phylum**	
*Proteobacteria*	84±3
*Actinobacteria*	9±1
*Planctomycetes*	3±1
Others	3±1
**Class**	
*Alphaproteobacteria*	72±4
*Actinobacteria*	10±1
*Betaproteobacteria*	8±1
Others	10±2
**Order**	
*Rhizobiales*	55±3
*Sphingomonadales*	14±2
*Actinomycetales*	11±1
Others	21±3
**Family**	
*Bradyrhizobiaceae*	26±3
*Rhizobiaceae*	17±6
*Sphingomonadaceae*	11±2
Others	47±4
**Genus**	
*Mesorhizobium*	14±2
*Bradyrhizobium*	11±1
*Streptomyces*	9±2
Others	66±5

a Others represents the sum of the taxonomic groups except the majour groups listed at each taxonoic level.

b The values represent means ± standard deviation (*n*=3).
