# Supplementary material for: Metagenomic Analysis of the Bacterial Community Associated with the Taproot of Sugar Beet
Source: Microbes Environ. 2015 Feb 14;30(1):63–9. doi: 10.1264/jsme2.ME14109 (PMC4356465; doi:10.1264/jsme2.ME14109)
Supplement: Supplementary file 1 [file 30_63_s1.pdf]

Table S1. Chemical characteristics of soils in the experimental fields

Experimental field	pH	P_2O_5 ($mg \cdot kg^{-1}$)	K_2O ($mg \cdot kg^{-1}$)	M_gO ($mg \cdot kg^{-1}$)	C_aO ($mg \cdot kg^{-1}$)	Total nitrogen (%)	NO_3-N ($mg \cdot kg^{-1}$)	NH_4-N ($mg \cdot kg^{-1}$)	Phosphate absorption coefficient	CEC ($mg \cdot kg^{-1}$)
Hokkaido	5.4	106	181	135	1,498	0.27	36	5.5	1,812	196
Yamagata	6.0	516	464	1,419	6,053	0.15	149	272	1,101	301

Table S2. Methanol dehydrogenase (MDH)-related sequence reads in the bacterial community of sugar beet taproot

	Type of MDH [a]	Length (bp)	Results of TBLASTN [b]															
			xoxF								mxaF							
			E-value	Identity (%)	Hit length	Hit start	Hit end	Query start	Query end	Hit frame	E-value	Identity (%)	Hit length	Hit start	Hit end	Query start	Query end	Hit frame
**NPK9**																		
HSSJVCC01DDEZ5	*xoxF*	744	2.E-100	75	588	1	588	350	551	1	1.E-66	50	663	1	663	351	573	1
HSSJVCC01EGHET	*xoxF*	712	3.E-77	74	474	1	474	31	188	-2	3.E-47	50	498	4	501	16	187	-2
HSSJVCC01EIJ9D	UD	908	3.E-76	74	474	1	474	31	188	-3	2.E-46	50	498	4	501	16	187	-3
HSSJVCC01CQL3E	UD	640	7.E-36	42	537	25	561	40	219	-2	3.E-34	42	549	46	594	23	212	-2
HSSJVCC01EG7FS	UD	708	1.E-13	41	315	378	692	447	551	-2	8.E-25	53	342	237	578	483	593	-2
HSSJVCC01E38BH [c]	UD	828	3.E-11	41	300	375	674	43	137	-2								
HSSJVCC01AIV7K [d]	UD	632									7.E-13	40	270	135	404	465	554	3
**NPK10**																		
HSSJVCC01ENAYA	*xoxF*	855	4.E-154	79	795	5	799	212	478	-3	4.E-76	52	747	44	790	215	457	-3
HSSJVCC01BYXFR	UD	944	1.E-86	45	933	11	943	122	441	2	2.E-91	47	942	2	943	113	434	2
HSSJVCC01BR07U	UD	771	6.E-68	45	765	3	767	277	542	3	2.E-57	40	762	3	764	277	538	3
HSSJVCC01CH5JZ [c]	UD	772	1.E-54	44	648	3	650	277	503	3								
HSSJVCC01C9LBB	UD	703	7.E-26	40	459	24	482	239	392	3	4.E-22	41	357	75	431	253	369	3
HSSJVCC01CTAAW	UD	753	7.E-15	46	357	6	362	485	600	3	6.E-25	51	351	6	356	482	595	3
HSSJVCC01D6QX0 [d]	UD	694									4.E-41	40	657	1	657	144	366	-2
**NPK12**																		
HSSJVCC01EID1Y	*xoxF*	829	5.E-129	66	825	3	827	197	473	-3	4.E-72	46	807	21	827	197	460	-3
HSSJVCC01D8JMI	*xoxF*	807	2.E-122	77	678	1	678	325	551	1	3.E-75	50	780	1	780	325	582	1
HSSJVCC01C9RDW	*xoxF*	704	1.E-108	74	618	12	629	314	520	-1	8.E-53	45	666	15	680	304	516	-1
HSSJVCC01CUNRH	*xoxF*	828	1.E-92	79	501	2	502	123	289	2	3.E-69	51	498	5	502	118	289	2
HSSJVCC01D35ZZ	*xoxF*	772	6.E-89	80	639	3	641	387	600	3	1.E-61	51	591	45	635	394	595	3
HSSJVCC01B9HLP	*xoxF*	678	5.E-88	69	591	1	591	22	215	-1	3.E-57	51	591	1	591	16	215	-1
HSSJVCC01BHF8J	*xoxF*	925	2.E-82	80	621	305	925	395	601	-1	1.E-60	52	594	314	907	394	595	-1
HSSJVCC01C9A3H	*xoxF*	664	4.E-77	80	450	3	452	387	537	3	2.E-46	51	405	45	449	394	533	3
HSSJVCC01ELEBG	*xoxF*	663	9.E-66	73	585	56	640	405	600	2	1.E-53	49	567	68	634	402	595	2
HSSJVCC01D5XQ9	UD	794	6.E-55	64	282	312	593	22	115	3	1.E-30	48	282	312	593	16	109	3
HSSJVCC01CJTS3	UD	863	6.E-49	50	552	312	863	22	206	3	8.E-39	51	330	312	641	16	125	3
HSSJVCC01B7T8N [c]	UD	711	2.E-47	40	702	5	706	50	306	-3								
HSSJVCC01DP43C	UD	742	4.E-41	47	594	109	702	404	600	-2	1.E-41	42	621	115	735	382	595	-2
HSSJVCC01DYIQQ	UD	616	5.E-32	42	456	124	579	22	172	1	3.E-27	41	456	124	579	16	172	1
HSSJVCC01CWEH8 [c]	UD	618	1.E-30	40	450	9	458	178	337	3								
HSSJVCC01CH9JT	UD	429	1.E-26	41	423	5	427	148	306	-3	8.E-30	42	423	5	427	148	306	-3
HSSJVCC01DVPUJ [c]	UD	515	2.E-17	41	411	102	512	420	551	-1								
HSSJVCC01CJS2K [d]	UD	808									5.E-44	40	711	6	716	177	424	3
HSSJVCC01DK6FC [d]	UD	620									5.E-12	40	261	167	427	467	553	2
HSSJVCC01DKZ3R [d]	UD	435									6.E-12	40	261	167	427	467	553	2

[a] Type of MDH was determined by the distance of clusters in a phylogenetic tree analysis. The UD means "undetermined" because a metagenome sequence read located in the out group of the trees of *xoxF* and *mxaF* genes.

[b] In this TBLASTN search, *xoxF* and *mxaF* genes in *Methylobacterium extorquens* AM1 were used as query sequences (YP 002962861 and ACS42169, respectively). The metagenome sequences were used as database.

[c] identified only by TBLASTN using an amino acid sequence of *xoxF* gene as query.

[d] identified only by TBLASTN using an amino acid sequence of *mxaF* gene as query.

Table S3. Detection frequency of the genes for siderophore in the bacterial community of sugar beet taproot

	ID of KEGG	Gene length [a]		Frequency per 10^5 reads [b]
		ID of UniRef	length (kbp)	
pchB ; isochorismate pyruvate lyase [EC:4.2.99.21]	K04782	UniRef50_Q51507	0.306	10 ± 7
dhbF ; nonribosomal peptide synthetase DhbF	K04780	UniRef50_C6U462	3.270	3 ± 0.6
entF ; enterobactin synthetase component F [EC:2.7.7.-]	K02364	UniRef50_P11454	3.882	1 ± 0.6
menF ; menaquinone-specific isochorismate synthase [EC:5.4.4.2]	K02552	UniRef50_A4SS27	1.356	0.8 ± 0.6
mbtG ; mycobactin lysine-N-oxygenase	K04793	UniRef50_Q7TYQ9	1.296	0.6 ± 0.8
mbtD ; mycobactin polyketide synthetase MbtD	K04791	UniRef50_S4ZBN7	3.102	0.5 ± 0.7
entE , 2,3-dihydroxybenzoate-AMP ligase [EC:2.7.7.58]	K02363	UniRef50_Q8XBV3	1.611	0.4 ± 0.6
mbtE ; mycobactin peptide synthetase MbtE	K04789	UniRef50_I2AEV7	5.031	0.4 ± 0.4
irp2 ; yersiniabactin nonribosomal peptide synthetase	K04784	UniRef50_P48633	6.108	0.3 ± 0.2
mbtA ; mycobactin salicyl-AMP ligase [EC:6.3.2.-]	K04787	UniRef50_P40871	1.620	0.3 ± 0.4
vibF ; nonribosomal peptide synthetase VibF	K12237	UniRef50_F2IQY7	7.254	0.2 ± 0.2
mbtB ; mycobactin phenyloxazoline synthetase	K04788	UniRef50_Q7TYQ4	4.245	0.1 ± 0.1
irp1 ; yersiniabactin nonribosomal peptide/polyketide synthase	K04786	UniRef50_A0A067HFK8	9.492	0.1 ± 0.1
entA ; 2,3-dihydro-2,3-dihydroxybenzoate dehydrogenase [EC:1.3.1.28]	K00216			ND
entB ; bifunctional isochorismate lyase / aryl carrier protein [EC:3.3.2.1]	K01252			ND
pchA ; salicylate biosynthesis isochorismate synthase [EC:5.4.4.2]	K01851			ND
entC ; isochorismate synthase [EC:5.4.4.2]	K02361			ND
vibH ; vibriobactin synthetase	K04778			ND
mbtI ; salicylate synthetase [EC:5.4.4.2 4.2.99.21]	K04781			ND
irp5 ; yersiniabactin salicyl-AMP ligase [EC:6.3.2.-]	K04783			ND
irp3 ; yersiniabactin synthetase, thiazolinyl reductase component	K04785			ND
mbtC ; mycobactin polyketide synthetase MbtC	K04790			ND
mbtF ; mycobactin peptide synthetase MbtF	K04792			ND
pchD ; pyochelin biosynthesis protein PchD	K12238			ND
pchE ; dihydroaeruginoic acid synthetase	K12239			ND
pchF ; pyochelin synthetase	K12240			ND
pchG ; pyochelin biosynthetic protein PchG	K12241			ND
mxcG ; nonribosomal peptide synthetase MxcG	K15653			ND
mxcL ; aminotransferase MxcL	K15681			ND

[a] Approximately gene length (kbp) was deduced by using the information of the UniProt Reference Clusters (UniRef) database. The gene length of the un-detected genes was not surveyed.

[b] ND = not detected. The values represent means ± standard deviation (n = 3).

Table S4. Taxonomic assingnment of β-1,3-glucanase related sequence reads.

Taxonomic group [a]	Frequency per 10^5 reads [b]
Bradyrhizobium	4 ± 2
Afipia	4 ± 4
Nitrobacter	4 ± 2
Rhodopseudomonas	1 ± 2
Bradyrhizobiaceae (Family) [c]	1 ± 0.8
Oligotropha	0.8 ± 1
Rhodovulum	0.8 ± 1
Chaetomium	0.5 ± 0.7
Neosartorya	0.5 ± 0.7
Sum	18 ± 6

[a] Gray indicates major taxonomic groups (*Mesorhizobium*, *Bradyrhizobium* and *Streptomyces*) at genus level in the bacterial community of sugar beet taproot.

[b] The values represent means ± standard deviation (*n* = 3).

[c] Taxonomic assignment of these sequence reads was not determined at genus level, but determined at family level by MEGAN.

Table S5. Taxonomic assingnment of quinoprotein glucose dehydrogenase (GDH) related sequence reads.

Taxonomic group [a]	Frequency per 10^5 reads [b]
Sphingobium	2 ± 0.7
Bradyrhizobium	1 ± 1
Chthoniobacter	0.9 ± 0.1
Rickettsiella	0.7 ± 1.0
Pseudomonas	0.7 ± 0.2
Rhizobium	0.6 ± 0.5
Sphingomonas	0.6 ± 0.5
Brevundimonas	0.6 ± 0.8
Ochrobactrum	0.6 ± 0.8
Agrobacterium	0.5 ± 0.4
Sinorhizobium	0.5 ± 0.4
Mesorhizobium	0.5 ± 0.7
Verrucomicrobium	0.5 ± 0.7
Sorangium	0.5 ± 0.4
Gemmata	0.3 ± 0.5
Alcanivorax	0.3 ± 0.4
Bilophila	0.3 ± 0.4
Chelativorans	0.3 ± 0.4
Citrobacter	0.3 ± 0.4
Microvirga	0.3 ± 0.4
Planctomyces	0.3 ± 0.4
Schlesneria	0.3 ± 0.4
Variovorax	0.3 ± 0.4
Halomonas	0.2 ± 0.3
Pirellula	0.2 ± 0.3
Robiginitalea	0.2 ± 0.3
Stenotrophomonas	0.2 ± 0.3
Terriglobus	0.2 ± 0.3
Blastopirellula	0.2 ± 0.3
Singulisphaera	0.2 ± 0.2
Tistrella	0.2 ± 0.2
Unknown	0.2 ± 0.2
Sum	15 ± 5

[a] Gray indicates major taxonomic groups (*Mesorhizobium* , *Bradyrhizobium* and *Streptomyces*) at genus level in the bacterial community of sugar beet taproot.

[b] The values represent means ± standard deviation (n = 3).

Table S6. Taxonomic assingnment of methanol dehydrogenase (MDH) related sequence reads.

Taxonomic group [a]	Frequency per 10^5 reads [b]
Bradyrhizobium	3 ± 2
Mesorhizobium	2 ± 0.6
Novosphingobium	0.9 ± 0.6
Rhodopseudomonas	0.7 ± 1
Azospirillum	0.6 ± 0.9
Afipia	0.6 ± 0.4
Thiorhodococcus	0.4 ± 0.6
Variovorax	0.4 ± 0.6
Burkholderia	0.3 ± 0.5
Curvibacter	0.3 ± 0.5
Methyloferula	0.2 ± 0.3
Pelagibaca	0.2 ± 0.3
Rhizobium	0.2 ± 0.3
Sum	10 ± 3

[a] Gray indicates major taxonomic groups (*Mesorhizobium*, *Bradyrhizobium* and *Streptomyces*) at genus level in the bacterial community of sugar beet taproot.

[b] The values represent means ± standard deviation ($n = 3$).

Table S7. Taxonomic assingnment of isochorismate pyruvate lyase (*pchB*) related sequence reads.

Taxonomic group	Frequency per 10^5 reads [a]
Sinorhizobium	5 ± 7
Pelagibacterium	3 ± 4
Pseudomonas	1 ± 2
Jannaschia	1 ± 2
Sum	10 ± 7

[a] The values represent means ± standard deviation (n = 3).

Table S8. Taxonomic assingnment of ACC deaminase related sequence reads.

Taxonomic group [a]	Frequency per 10^5 reads [b]
Rhizobium	2 ± 3
Bradyrhizobium	2 ± 1
Agrobacterium	1 ± 0.9
Pseudomonas	1 ± 0.9
Streptomyces	0.7 ± 1
Protochlamydia	0.6 ± 0.8
Variovorax	0.6 ± 0.8
Collimonas	0.4 ± 0.6
Phyllobacterium	0.4 ± 0.6
Sum	9 ± 6

[a] Gray indicates major taxonomic groups (*Mesorhizobium* , *Bradyrhizobium* and *Streptomyces*) at genus level in the bacterial community of sugar beet taproot.

[b] The values represent means ± standard deviation (n = 3).

Table S9. Taxonomic assingnment of chitinase related sequence reads.

Taxonomic group [a]	Frequency per 10^5 reads [b]
Streptomyces	0.9 ± 1.2
Chaetomium	0.6 ± 0.8
Geobacter	0.6 ± 0.8
Aspergillus	0.5 ± 0.7
Stenotrophomonas	0.5 ± 0.7
Trichophyton	0.5 ± 0.7
Rhodopirellula	0.4 ± 0.6
Stanieria	0.4 ± 0.6
Amycolatopsis	0.3 ± 0.4
Bradyrhizobium	0.3 ± 0.4
Grosmannia	0.3 ± 0.4
Magnaporthe	0.3 ± 0.4
Myceliophthora	0.3 ± 0.4
Mycobacterium	0.3 ± 0.4
Xanthomonas	0.3 ± 0.4
Sum	6 ± 4

[a] Gray indicates major taxonomic groups (*Mesorhizobium* , *Bradyrhizobium* and *Streptomyces*) at genus level in the bacterial community of sugar beet taproot.

[b] The values represent means ± standard deviation (n = 3).

Table S10. Detection frequency of the genes for N metabolism in the bacterial community of sugar beet taproot

	ID of KEGG	Gene length [a]		Frequency per 10^5 reads [b]
		ID of UniRef	length (kbp)	
glnA ; glutamine synthetase [EC:6.3.1.2]	K01915	UniRef50_A0R079	1.437	5 ± 0.6
cynT , can; carbonic anhydrase [EC:4.2.1.1]	K01673	UniRef50_Q9I262	0.663	4 ± 0.6
ncd2 , *npd* ; nitronate monooxygenase [EC:1.13.12.16]	K00459	UniRef50_F8GQA6	1.254	3 ± 0.4
gudB, rocG; glutamate dehydrogenase [EC:1.4.1.2]	K00260	UniRef50_B2RKJ1	1.338	3 ± 0.2
gltD; glutamate synthase (NADPH/NADH) small chain [EC:1.4.1.13 1.4.1.14]	K00266	UniRef50_P9WN18	1.467	2 ± 0.3
gltB; glutamate synthase (NADPH/NADH) large chain [EC:1.4.1.13 1.4.1.14]	K00265	UniRef50_Q05755	4.548	2 ± 0.3
nirK ; nirnitrite reductase (NO-forming) [EC:1.7.2.1]	K00368	UniRef50_P81445	0.993	1 ± 0.8
nirB ; nitrite reductase (NADH) large subunit [EC:1.7.1.15]	K00362	UniRef90_A6UI45	2.463	1 ± 0.3
cynS ; cyanate lyase [EC:4.2.1.104]	K01725	UniRef50_A2SLJ8	0.444	1 ± 0.7
E1.4.1.4, gdhA; glutamate dehydrogenase (NADP+) [EC:1.4.1.4]	K00262	UniRef50_B2RKJ1	1.338	0.9 ± 0.2
nasA ; assimilatory nitrate reductase catalytic subunit [EC:1.7.99.4]	K00372	UniRef50_E1VMR6	2.820	0.9 ± 0.2
nirD ; nitrite reductase (NADH) small subunit [EC:1.7.1.15]	K00363	UniRef50_P0A230	0.327	0.8 ± 0.7
E3.5.1.49; formamidase [EC:3.5.1.49]	K01455	UniRef50_Q887D9	1.017	0.7 ± 0.09
E3.5.5.1; nitrilase [EC:3.5.5.1]	K01501	UniRef50_F8DUB5	0.990	0.7 ± 0.3
GLUD1_2, *gdhA* ; glutamate dehydrogenase (NAD(P)+) [EC:1.4.1.3]	K00261	UniRef50_Q0SJ78	1.290	0.6 ± 0.3
napA ; periplasmic nitrate reductase NapA [EC:1.7.99.4]	K02567	UniRef50_B1Y6A6	2.556	0.6 ± 0.1
norB ; nitric oxide reductase subunit B [EC:1.7.2.5]	K04561	UniRef50_P98008	1.425	0.5 ± 0.1
norC ; nitric oxide reductase subunit C	K02305	UniRef50_O06844	0.444	0.3 ± 0.5
arcC ; carbamate [EC:2.7.2.2]	K00926	UniRef50_Q8FK51	0.894	0.3 ± 0.3
nirA ; ferredoxin-nitrite reductase [EC:1.7.7.1]	K00366	UniRef50_Q89LH3	1.878	0.3 ± 0.07
E1.4.7.1; glutamate synthase (ferredoxin) [EC:1.4.7.1]	K00284	UniRef50_P09831	4.461	0.3 ± 0.09
narG ; nitrate reductase alpha subunit [EC:1.7.99.4]	K00370	UniRef50_P9WJQ2	3.699	0.3 ± 0.05
narJ ; nitrate reductase delta subunit	K00373	UniRef50_P0AF27	0.711	0.2 ± 0.05
nosZ ; nitrous-oxide reductase [EC:1.7.2.4]	K00376	UniRef50_Q8FX16	1.920	0.2 ± 0.09
narH ; nitrate reductase beta subunit [EC:1.7.99.4]	K00371	UniRef50_Q83RN5	1.539	0.2 ± 0.2
nasB ; assimilatory nitrate reductase electron transfer subunit [EC:1.7.99.4]	K00360	UniRef50_A0JUE0	1.614	0.1 ± 0.1
narI ; nitrate reductase gamma subunit [EC:1.7.99.4]	K00374	UniRef50_P0AF33	0.681	0.1 ± 0.2
nrfA ; nitrite reductase (cytochrome c-552) [EC:1.7.2.2]	K03385	UniRef50_Q4QLL8	1.617	0.06 ± 0.05
GLT1; glutamate synthase (NADPH/NADH) [EC:1.4.1.13 1.4.1.14]	K00264	UniRef50_Q0DG35	6.567	0.01 ± 0.01
NRT, narnrtP, *nasA* ; MFS transporter, NNP family, nitrate/nitrite transporter	K02575			ND
nrtA, nasF, cynA ; nitrate/nitrite transport system substrate-binding protein	K15576			ND
nrtB, nasE, cynB ; nitrate/nitrite transport system permease protein	K15577			ND
nrtC, nasD ; nitrate/nitrite transport system ATP-binding protein [EC:3.6.3.-]	K15578			ND
nrtD, cynD ; nitrate/nitrite transport system ATP-binding protein	K15579			ND
narB ; ferredoxin-nitrate reductase [EC:1.7.7.2]	K00367			ND
NR; nitrate reductase (NAD(P)H) [EC:1.7.1.1 1.7.1.2 1.7.1.3]	K10534			ND
napB ; cytochrome c-type protein NapB	K02568			ND
NIT-6; nitrite reductase (NAD(P)H) [EC:1.7.1.4]	K17877			ND
nrfH ; cytochrome c nitrite reductase small subunit	K15876			ND
nirS ; nitrite reductase (NO-forming) / hydroxylamine reductase [EC:1.7.2.1 1.7.99.1]	K15864			ND
CYP55; fungal nitric oxide reductase [EC:1.7.1.14]	K15877			ND
nifD ; nitrogenase molybdenum-iron protein alpha chain [EC:1.18.6.1]	K02586			ND
nifnitrogenase molybdenum-iron protein beta chain [EC:1.18.6.1]	K02591			ND
nifH ; nitrogenase iron protein NifH [EC:1.18.6.1]	K02588			ND
anfG ; nitrogenase delta subunit [EC:1.18.6.1]	K00531			ND
pmoA-amoA; methane/ammonia monooxygenase subunit A [EC:1.14.18.3 1.14.99.39]	K10944			ND
pmoB-amoB; methane/ammonia monooxygenase subunit B	K10945			ND
pmoC-amoC; methane/ammonia monooxygenase subunit C	K10946			ND
hcp ; hydroxylamine reductase [EC:1.7.99.1]	K05601			ND
hao ; hydroxylamine dehydrogenase [EC:1.7.2.6]	K10535			ND
GDH2; glutamate dehydrogenase [EC:1.4.1.2]	K15371			ND
CPS1; carbamoyl-phosphate synthase (ammonia) [EC:6.3.4.16]	K01948			ND
E4.2.1.1; carbonic anhydrase [EC:4.2.1.1]	K01672			ND
cah ; carbonic anhydrase [EC:4.2.1.1]	K01674			ND

[a] Approximately gene length (kbp) was deduced by using the information of the UniProt Reference Clusters (UniRef) database. The gene length of the un-detected genes was not surveyed.

[b] ND = not detected. The values represent means ± standard deviation (n = 3).

```
          5         15         25         35         45         55         65         75         85         95
XoxF     -------MRA VHLLALGAGL AAASPALANE SVLKGVANPA EQVLQTVDYA NTRYSKLDQI NASNVKNLQV AWTFSTGVLR GHEGSPLVVG NIMYVHTPFP
MxaF     MSRFVTSVSA LAMLALAP-A ALSSGAYAND KLVELSKSDD NWVMPGKNYD SNNFSDLKQI NKGNVKQLRP AWTFSTGLLN GHEGAPLVVD GKMYIHTSFP
                  *   ***     *  * * **              *     *      * * ** *  *** *  ******* *  **** ****    ** ** **

               105        115        125        135        145        155        165        175        185        195
XoxF     NIVYALDLDQ GAKIVWKYEP KQDPSVIPVM CCDTVNRGLA YADG------ AILLHQADTT LVSLDAKSGK VNWSVKNGDP SKGETNTATV LPVKDKVIVG
MxaF     NNTFALGLDD PGTILWQDKP KQNPAARAVA CCDLVNRGLA YWPGDGKTPA LILKTQLDGN VAALNAETGE TVWKVENSDI KVGSTLTIAP YVVKDKVIIG
         *   ** **    * *   * ** *    * *** ****** *  *       **  * *     * *  *   * * * *   * * *      ****** *

               205        215        225        235        245        255        265        275        285        295
XoxF     ISGGEFGVQC HVTAYDLKSG KKVWRGYSIG PDDQLIVDPE KTTSLGKPIG KDSSLKTWEG DQWKTGGGCT WGWFSYDPKL DLMYYGSGNP STWNPKQRPG
MxaF     SSGAELGVRG YLTAYDVKTG EQVWRAYATG PDKDLLLASD FNIKNPHYGQ KGLGTGTWEG DAWKIGGGTN WGWYAYDPGT NLIYFGTGNP APWNETMRPG
          ** * **     **** * *   *** *  * ** *                *     **** * ** ***  ***  ***    * * * ***  **   ***

               305        315        325        335        345        355        365        375        385        395
XoxF     DNKWSMTIWA RNPDTGMAKW VYQMTPHDEW DFDGINEMIL TDQK-FDGKD RPLLTHFDRN GFGYTLDRAT GEVLVAEKFD PVVNWATKVD LDKGSKTYGR
MxaF     DNKWTMTIFG RDADTGEAKF GYQKTPHDEW DYAGVNVMML SEQKDKDGKA RKLLTHPDRN GIVYTLDRTD GALVSANKLD DTVNVFKSVD LKTG-----Q
         **** ***   *  *** **   ** ****** *  * * * *   **  ***  * **** *** *  *****    *    * * *   **    ** *  *

               405        415        425        435        445        455        465        475        485        495
XoxF     PLVVSKYSTE QNGEDVNSKG ICPAALGTKD QQPAAFSPKT GLFYVPTNHV CMDYEPFRVT YTPGQPYVGA TLSMYPAPG- ---SHGGMGN FIAWDNLQGK
MxaF     PVRDPEYGTR ---MDHLAKD ICPSAMGYHN QGHDSYDPKR ELFFMGINHI CMDWEPFMLP YRAGQFFVGA TLNMYPGPKG DRQNYEGLGQ IKAYNAITGD
         *     * *     *   * *** * *    *     **   **    **  *** ***    *  **  *** ** *** *         * *    *     *

               505        515        525        535        545        555        565        575        585        595
XoxF     IKWSNPEQFS AWGGALATAG DVVFYGTLEG FLKAVDSKTG KELYKFKTPS GIIGNVMTYE HKGKQHVAVL SGVGGWAGIG LAAGLTDPNA GLGAVGGYAA
MxaF     YKWEKMERFA VWGGTMATAG DLVFYGTLDG YLKARDSDTG DLLWKFKIPS GAIGYPMTYT HKGTQYVAIY YGVGGWPGVG LVFDLADPTA GLGAVGAFKK
          **   * *   ***  **** * ****** *  *** ** **    * *** ** * **  ***  *** * **    ***** * * *   * ** * ******

               605        615        625        635
XoxF     LSSYTNLGGQ LTVFSLPNN- ---------- -----
MxaF     LANYTQMGGG VVVFSLDGKG PYDDPNVGEW KSAAK
         *  **  **    ****
```

Figure S1. Amino acid sequence similarity between *xoxF* and *mxaF* genes in *Methylobacterium extorquens* AM1. The global alignment analysis was performed by ClustalW program on the Bioedit software (http://www.mbio.ncsu.edu/bioedit/bioedit.html).The consensus sequence was indicated by asterisk mark. BLASTP analysis revealed that the XoxF protein had 49% identities and 65% positives with the MxaF protein.

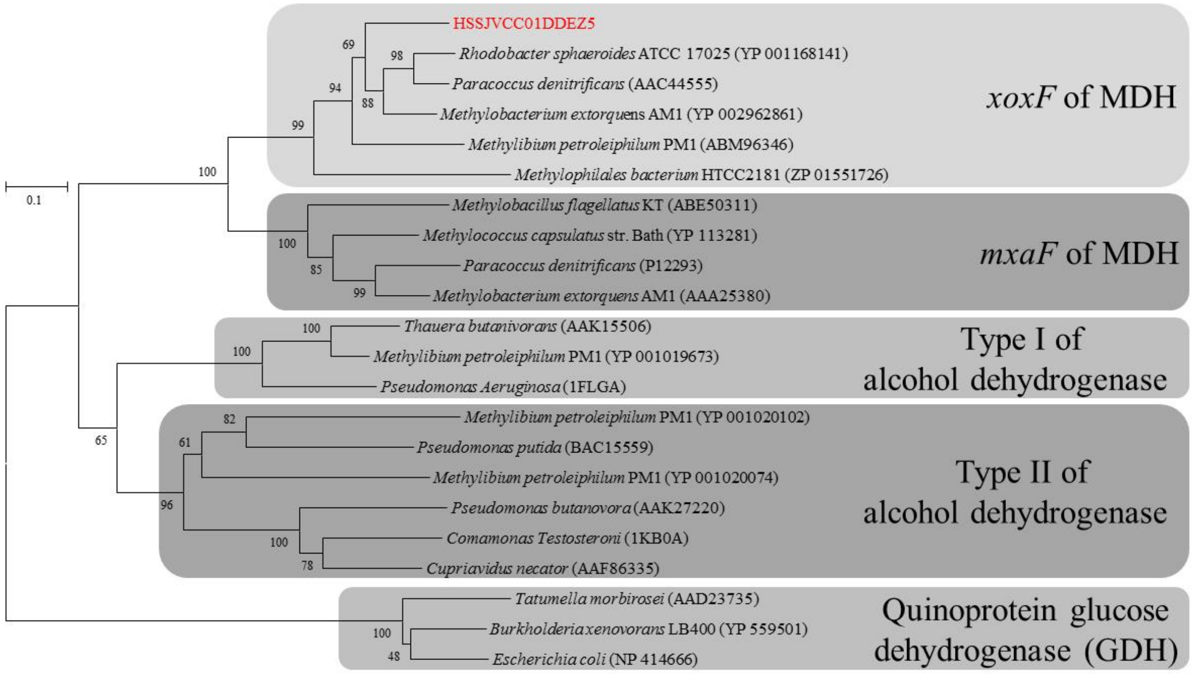

Figure S2. Phylogenetic distance tree analysis of methanol dehydrogenase (MDH)-related sequence reads. TBLASTN search found 34 reads of MDH in the metagenome of the bacterial community of sugar beet taproot. The MDH type (*xoxF* or *mxaF*) of these reads was determined by individual phylogenetic distance tree analyses. "HSSJVCC01DDEZ5" (in red) is a sequence read in sample NPK9. This read is located in the *xoxF* group of MDH. GenBank accession numbers and RefSeq numbers for reference sequences (quinoprotein alcohol dehydrogenase) are shown in parentheses. The scale bar represents 0.1 substitutions per site. The numbers at the nodes show the numbers out of 1000 bootstrap tests.

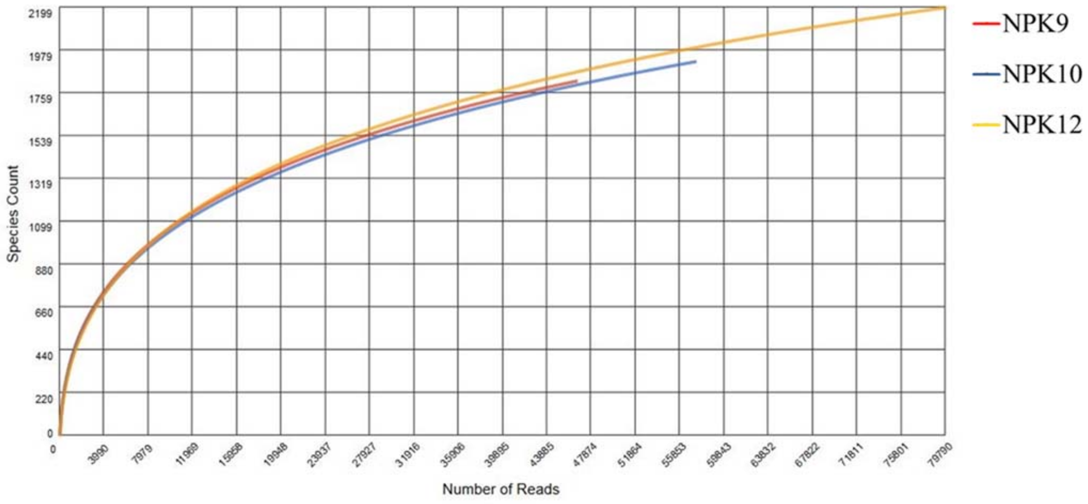

Figure S3. Rarefaction curve of the metagenomic DNA samples (samples NPK9, NPK10 and NPK12). The rarefaction curve was described by using the Metagenomics RAST server (MG-RAST; http://metagenomics.anl.gov). The data was compared to the non-redundant multi-source protein annotation database (M5NR) using a maximum e-value of $1e^{-10}$, a minimum identity of 60 %, and a minimum alignment length of 15 measured in amino acid for protein and bp for RNA databases. Alpha diversity of the metagenomic DNA samples (samples NPK9, NPK10 and NPK12) were 191, 186 and 211, respectively.

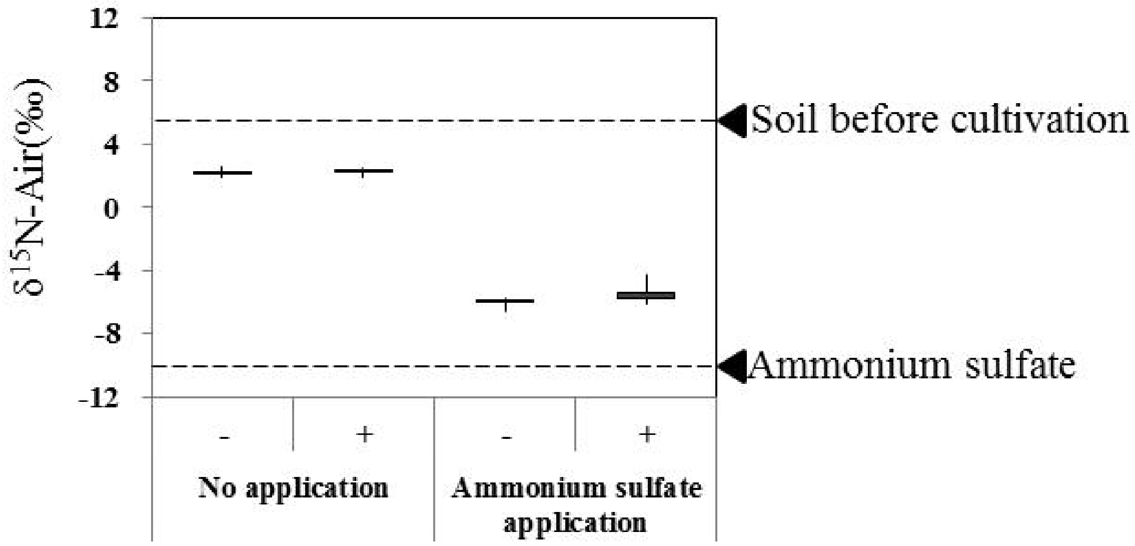

Figure S4. δ^{15}N-air (‰) in the roots of sugar beet exposed to $^{15}N_2$. Plants were grown with or without ammonium sulfate application. Harvested roots were exposed (+) to $^{15}N_2$ for 24 h. Unexposed roots were used as a negative control (–). Dotted lines show the δ^{15}N-air values of soil before cultivation (5.477 ‰) and of ammonium sulfate (-9.863 ‰).
